# Transversus Abdominus Plane Block for Laparoscopic Sleeve Gastrectomy—A Systematic Review and Meta-analysis of Randomized Clinical Trials

**DOI:** 10.1007/s11695-025-08166-z

**Published:** 2025-09-18

**Authors:** Matthew G. Davey, John C. Conneely, Jarlath C. Bolger, William B. Robb, Noel E. Donlon

**Affiliations:** 1 Royal College of Surgeons in Ireland, Dublin, Ireland; 2 Mater Misericordiae University Hospital, Dublin, Ireland; 3 Beaumont Hospital, Dublin, Ireland; 4 St. James’s Hospital, Dublin, Ireland

**Keywords:** Laparoscopic sleeve gastrectomy, Transversus abdominus plane block, TAP block

## Abstract

**Background:**

Transversus abdominus plane (TAP) blocks have become increasingly popular, due to a perceived reduction in post-operative pain following laparoscopic surgery. Their value following sleeve gastrectomy remains unclear.

**Objectives:**

To perform a systematic review and meta-analysis of randomized clinical trials (RCTs) evaluating the efficacy of TAP block in patients undergoing laparoscopic sleeve gastrectomy.

**Setting:**

Integration of data from bariatric surgery units across the world.

**Methods:**

A systematic review was performed as per PRISMA guidelines. Meta-analysis was performed using Review Manager v5.4.

**Results:**

Eleven RCTs including 776 patients were included with 338 randomized to TAP block (50.0%). A non-significant equipoise was observed between groups for mean age, gender, body mass indices, and American Society of Anesthesiologists grades (all *P* > 0.050). At meta-analyses, patients receiving TAP block had significantly reduced post-operative visual analogue scores (VAS) at 0–60 min (mean difference (MD), − 1.23; 95% confidence interval (CI), − 1.87 to − 0.58; *P* < 0.001), 2 h (MD, − 1.78; 95% CI, − 3.28 to − 0.27; *P* < 0.001), 4 h (MD, − 1.00; 95% CI, − 1.24 to − 0.76; *P* < 0.001), 6 h (MD, − 1.58; 95% CI, − 2.46 to − 0.69; *P* < 0.001), 12 h (MD, − 1.13; 95% CI, − 1.80 to − 0.46; *P* = 0.001), and 24 h (MD, − 0.77; 95% CI − 1.42 to − 0.12; *P* < 0.001) respectively. At meta-analysis, a non-significant difference was observed for breakthrough analgesia consumption, time to rescue analgesia, post-operative nausea and vomiting, time to ambulation, length of stay, and post-operative complications. Patient satisfaction scores were significantly in favour of TAP block (MD, 0.88; 95% CI, 0.49–1.28; *P* < 0.001).

**Conclusion:**

TAP block significantly reduced post-operative pain and improved patient satisfaction following sleeve gastrectomy. TAP block should be considered for patients undergoing this procedure, should expertise allow.

**Supplementary Information:**

The online version contains supplementary material available at 10.1007/s11695-025-08166-z.

## Introduction

Contemporary bariatric surgery has evolved such that minimally invasive surgery has come into vogue in recent times [1, 2]. This change is predominantly due to enhanced recovery times observed following minimally invasive approaches, due to the decreased post-operative pain and morbidity associated with smaller surgical incisions performed for minimally invasive surgery [3]. Emerging data indicates that patients with elevated body habitus may be subject to increased pain following surgery [4, 5], leading to the investigation and deployment of multimodal analgesic strategies to combat this negative sequalae of bariatric surgery [6]. Similar to the ethos developed within the context of enhanced recovery after bariatric surgery (ERABS) initiatives [7], the optimization of post-operative analgesia protocols is currently at the epicenter of efforts to achieve the “same day” 12-h discharges from hospital following bariatric surgery, with success rates in excess of 90% observed when these operations are performed in centers with such expertise [8, 9].

The peri-operative administration of transversus abdominus plane (TAP) blocks following several minimally invasive surgeries have become popularized in recent decades, largely due data from several randomized clinical trials (RCTs) and subsequent meta-analyses demonstrating their pragmatism in reducing post-operative pain compared to infiltration of local anesthetic into wounds, as is recognized as standard practice [10, 11]. Performing a TAP block involves the direct administration of local anesthesia into the potential space between the internal oblique and rectus abdominus musculature in the anterior abdominal wall [12], with the ambition of reducing post-operative pain, as well as facilitating a reduction in respiratory, thromboembolic, and other complications in the post-operative setting [13]. Therefore, these positive implications somewhat justify the use of TAP block in certain instances [10, 11, 14]; however, the clinical utility of TAP block in the peri-operative setting in patients undergoing minimally invasive sleeve gastrectomy remains less clear. Accordingly, the aim of the current study was to perform a systematic review and meta-analysis using solely RCT data to determine the impact of TAP block on outcomes following minimally invasive sleeve gastrectomy.

## Methods

This systematic review was conducted in accordance to the “Preferred Reporting Items for Systematic Reviews and Meta-Analyses” (PRISMA). This study was prospectively registered with the International Prospective Register of Systematic Reviews (PROSPERO–CRD42024622424). Local institutional ethical review and approval was not required for this study as it is a review of the currently available published evidence.

### Population, Intervention, Comparison Outcome Framework

Using the Population, Intervention, Comparison (PICO) framework [15], the aspects the authors wished to address were as follows:

- Population: Adult patients aged 18 years or older who were indicated to undergo minimally invasive sleeve gastrectomy in the RCT setting,
- Intervention: Any patient randomized to receive TAP block,
- Comparison: Any patient randomized to receive control.
- Outcomes:
  - The primary outcomes of interest included visual analogue score (VAS) measurements performed at 0-60 minutes, 2-hours, 4-hours, 6-hours, 12- hours, 24- hours, and 48-hours post-operatively.
  - The secondary outcomes of interest were rescue opioid consumption, time to first breakthrough opioid consumption (measured in hours), post-operative nausea and vomiting (PONV), time to ambulation (measured in hours), length of hospital stay (LOS, measured in days), complications, and patient satisfaction scores (PSS).

### Search Strategy

Two independent reviewers performed a systematic, electronic search of the PUBMED, EMBASE, and Cochrane library databases for relevant studies. This search included the search terms (sleeve gastrectomy) and (TAP block) which were linked by the Boolean operator “AND,” as described. Included studies were limited to the English language and were not restricted by year of publication. All duplicates were manually removed, before title were screened, and studies considered appropriate had their abstracts and/or full text reviewed. Retrieved studies were reviewed to ensure inclusion criteria were met for the primary outcome measure. In cases of discrepancies in opinion among reviewers as to study eligibility, the senior author was asked to arbitrate. The initial search was performed on the 1^st^ of November 2022 and the final search was performed on the 2^nd^ of December 2024.

### Eligibility Criteria

Studies were considered for inclusion if they were of prospective, randomized design comparing outcomes relevant to TAP block versus control in the laparoscopic sleeve gastrectomy peri-operative setting. Studies failing to fulfil this criteria were deemed ineligible and excluded from this study.

### Data Extraction

The following data were extracted and collected from retrieved studies meeting inclusion criteria: (1) first author name, (2) year of publication, (3) study design, (4) country of origin, (5) number of patients, (6) number of patients randomized to TAP and control respectively, (7) mean patient age of patients in each group, and (8) data pertaining to the aforementioned primary and secondary outcomes of interest.

### Statistical Analysis

Descriptive statistics were used to determine associations between TAP block and control and the primary and secondary outcomes of interest, as appropriate. This involved the use of the Fischer’s Exact (†) and independent samples *t*-tests (‡), where appropriate [16]. All tests of significance were two tailed with *P* < 0.050 representing statistical significance. Descriptive statistics were performed using the Statistical Package for Social Sciences (SPSS) version 26 (International Business Machines Corporation, Armonk, New York).

Thereafter, relevant outcomes for patients randomized to TAP or control were expressed as dichotomous, reported as odds ratios (ORs) with their corresponding 95% confident intervals (CIs), following estimation using the Mantel–Haenszel method. Continuous data was analysed using inverse variance methodology, with mean differences (MD) and CIs reported. Given the heterogeneity anticipated via including trials from surgical research institutions across the world, random effects models were applied irrespective of reported heterogeneity (*I*^*2*^). Meta-analyses were performed using Review Manager (RevMan), Version 5.4 (Nordic Cochrane Centre, Copenhagen, Denmark).

### Risk of Bias Assessment

As all included studies were of prospective, randomized design, methodology assessment was undertaken using the Risk of Bias 2.0 (ROB2) Assessment [17].

## Results

### Literature Search

**Fig. 1:**
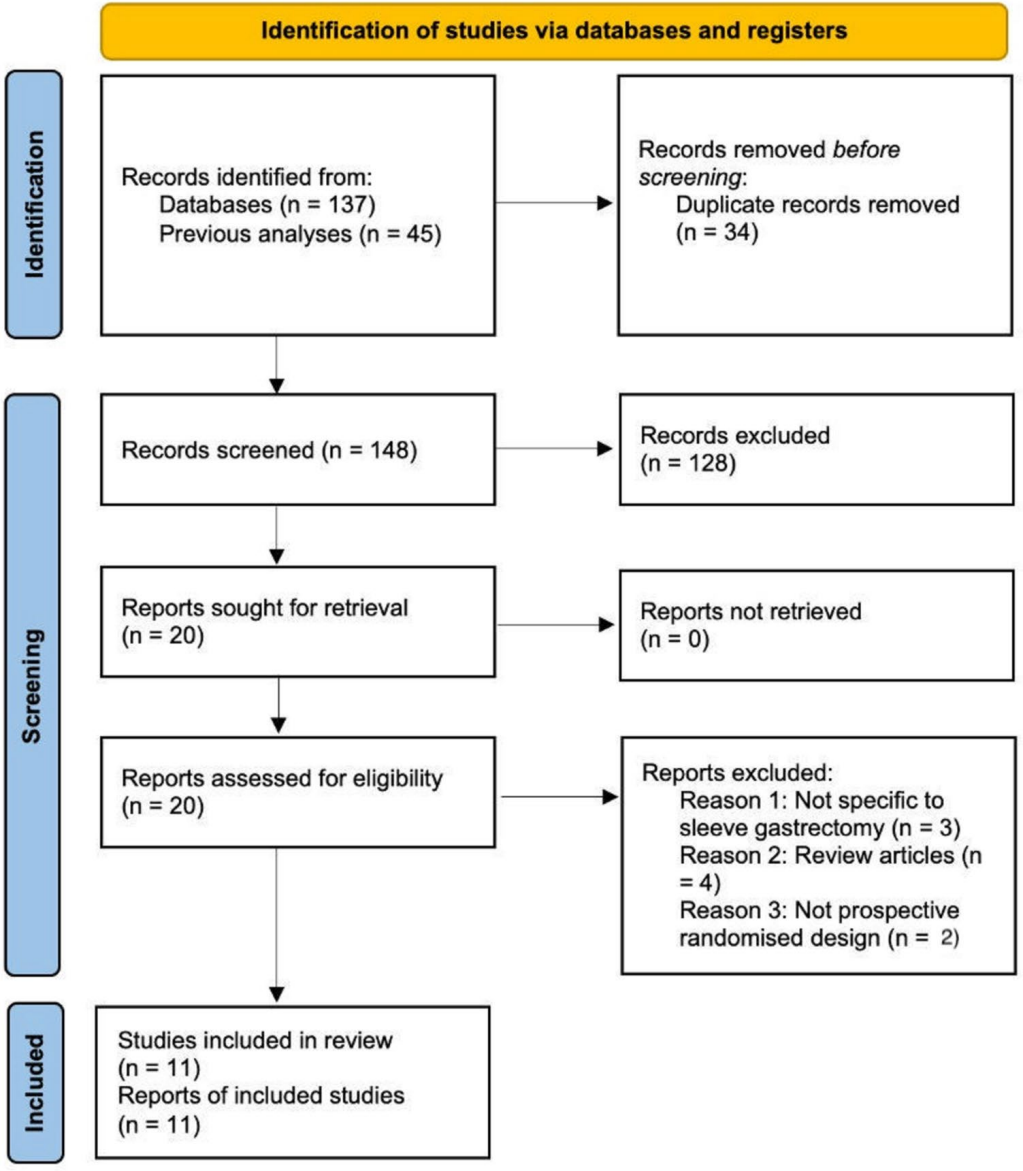
PRISMA flowchart demonstrating the systematic search process

In total, 182 articles were identified before 34 duplicate articles were excluded. Thereafter, 148 study titles were screened, before 20 abstracts were reviewed for relevance. All 20 of these studies were eligible for full-text review and 11 of RCTs met the eligibility criteria and were included in this systematic review and meta-analysis [18–28] (Fig. 1).

### Study Characteristics

All 11 RCTs reported outcomes in relation to patients indicated to undergo laparoscopic sleeve gastrectomy who were randomized to either TAP block or control (100.0%, 11/11) [18–28]. Publication dates of included studies ranged from 2014 to 2024. Study data for the 11 included RCTs are depicted in Table 1 while peri-operative information is outlined in Supplementary Material 1.

**Table 1:** Study data from the 11 included prospective, randomised clinical trials

Author	Year	Study design	Country	Inclusion criteria	Number	N TAP	N Control
Abdelhamid	2020	RCT	Egypt	Aged 18–59, ASA II/III, BMI > 40	44	22	22
Alver	2023	RCT	Turkey	Aged 18–65, ASA 1/II, BMI > 35	48	24	24
Cataldo	2024	RCT	Italy	Aged 18–65, with a BMI > 35 with one comorbidity related to obesity or BMI > 40	110	54	56
Hussein	2023	RCT	Egypt	Aged 25–60, with a BMI 35–45 with ASA II	30	15	15
Ibrahim	2014	RCT	Egypt	Aged > 18, with a BMI > 35 with ASA II/III	42	21	21
Mittal	2018	RCT	India	Aged 18–60, with a BMI > 32.5 with one comorbidity related to obesity or BMI > 35	60	30	30
Okut	2022	RCT	Turkey	BMI > 35 with one comorbidity related to obesity or BMI > 40	60	30	30
Saber	2018	RCT	USA	Aged 18–65, with ASA II/III	60	30	30
Sherif	2013	RCT	Egypt	Aged > 18, with BMI > 35 with ASA I/II/III	95	48	47
Xue	2022	RCT	China	Aged 18–65, ASA I/II, with a BMI > 35 with one comorbidity related to obesity or BMI > 40	156	78	78
Zhou	2024	RCT	China	Aged > 18, with ASA I/II/III	71	36	35

*N* number, *TAP* transversus abdominus plane block, *RCT* randomised clinical trial, *BMI* body mass index, *ASA* American college of Anesthesiologists

### Patient Characteristics

Overall, data from 776 patients was included, of whom 338 were randomized to TAP block (50.0%) and 338 to the control group (50.0%) respectively. For patients randomized to the TAP block and control groups, a non-significant difference was observed with respect to mean patient age (‡), gender (†), mean body mass indices (BMI) (‡), and American Society of Anesthesiologists (ASA) grade (†) (all *P* > 0.050).

### Visual Analogue Scores

**Fig. 2:**
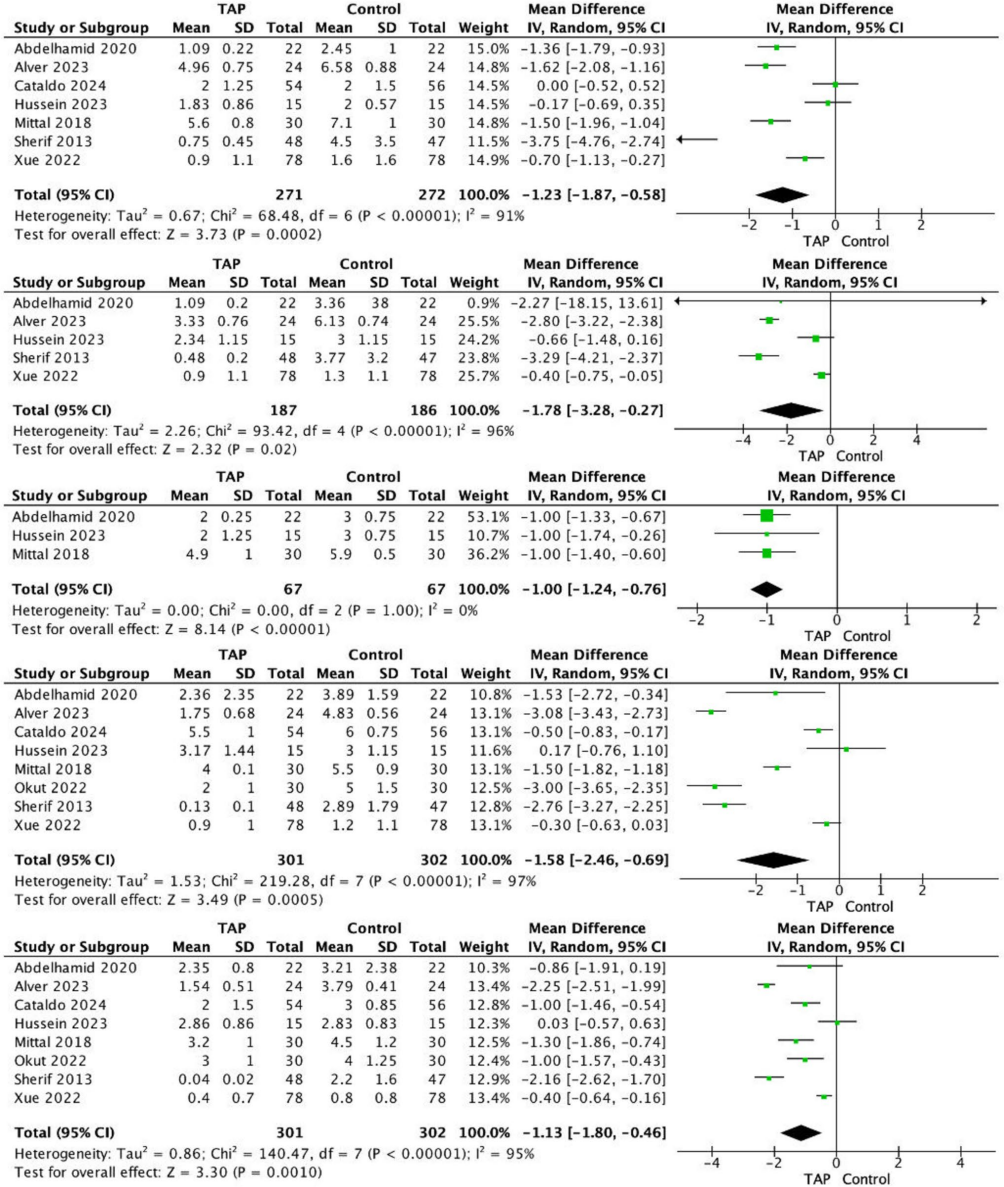
Visual Analogue scores for patients receiving transversus abdominus plane block compared to controls at (**A**) 0–60 min, **B** 2 h, **C** 4 h, **D** 6 h, and (**E**) 12 h following laparoscopic sleeve gastrectomy

**Fig. 3:**
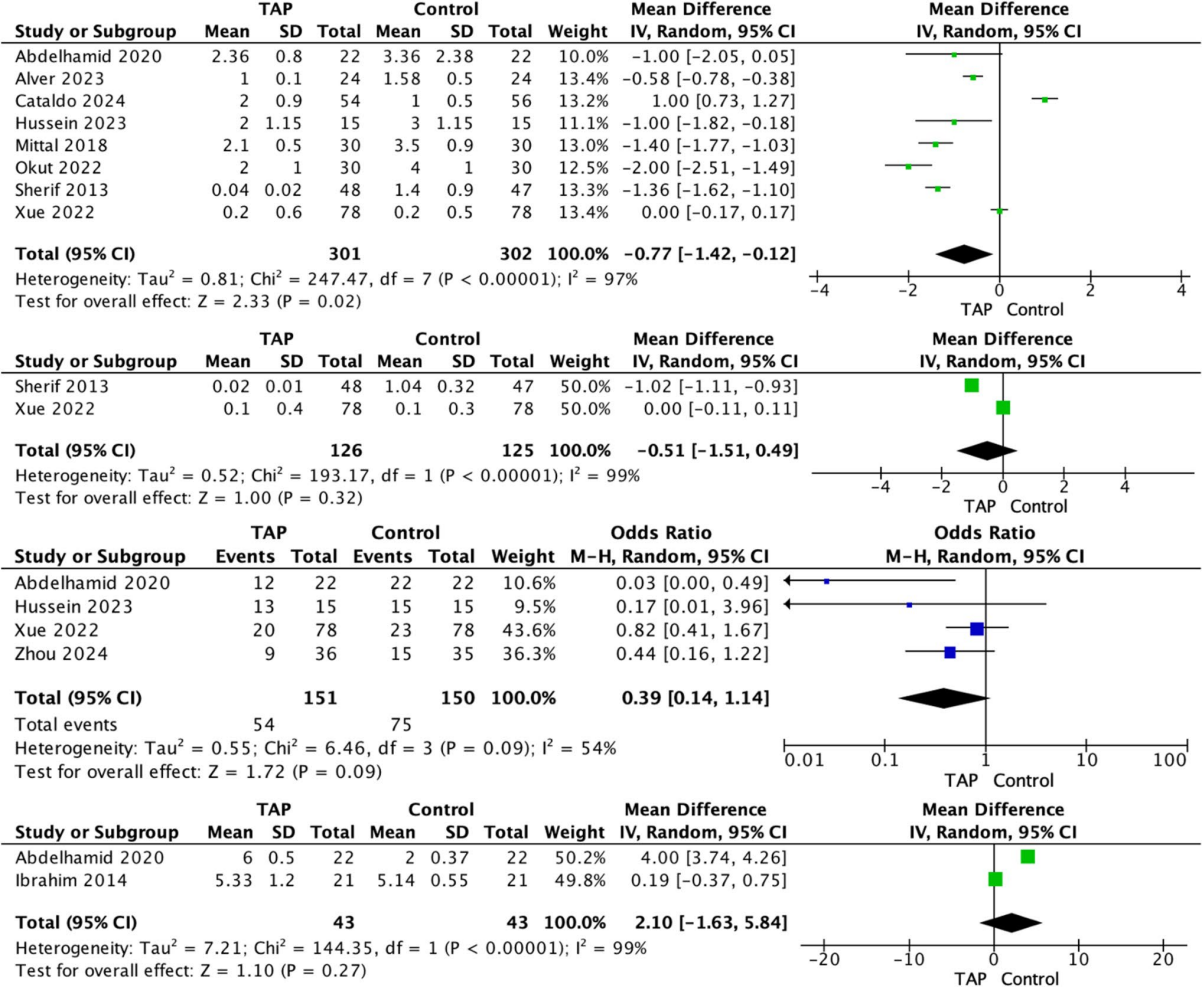
Visual Analogue scores for patients receiving transversus abdominus plane block compared to controls at (**A**) 24 h and (**B**) 48 h following laparoscopic sleeve gastrectomy. The proportion of patients who received rescue analgesia is demonstrated in (**C**) while (**D**) represents the time to rescue analgesia request

VAS scores were reported in all 11 included RCTs [18–28]. At meta-analyses, patients receiving TAP block had significantly reduced post-operative VAS at 0–60 min (MD, − 1.23; 95% CI, − 1.87 to − 0.58; *P* < 0.001) (Fig. 2A), 2 h (MD, − 1.78; 95% CI, − 3.28 to − 0.27; *P* < 0.001) (Fig. 2B), 4 h (MD, − 1.00; 95% CI, − 1.24 to − 0.76; *P* < 0.001) (Fig. 2C), 6 h (MD, − 1.58; 95% CI, − 2.46 to − 0.69; *P* < 0.001) (Fig. 2D), 12 h (MD, − 1.13; 95% CI, − 1.80 to − 0.46; *P* = 0.001) (Fig. 2E), and 24 h (MD, − 0.77; 95% CI − 1.42 to − 0.12; *P* < 0.001) (Fig. 3A) respectively. A non-significant difference observed at meta-analysis with respect to VAS scores at 48 h (MD, − 0.51; 95% CI − 1.51 to 0.49; *P* = 0.020) (Fig. 3B).

### Breakthrough Opioid Consumption

Five RCTs reported the consumption of breakthrough opioid use and the time taken until breakthrough opioid administration [18, 21, 22, 27, 28]. Overall, 35.8% of patients receiving TAP block required breakthrough opioid analgesia (54/151), compared to 49.7% in the control group (75/151) (*P* = 0.020, †). At meta-analysis, a non-significant difference was observed in relation to breakthrough opioid consumption for patients randomized to TAP and control groups respectively (OR, 0.39; 95% CI, 0.14–1.14; *P* = 0.090) (Fig. 3C). There was also a non-significant difference was observed in relation to time to breakthrough opioid consumption (MD, 2.10; 95% CI, − 1.63–5.84; *P* = 270) (Fig. 3D).

### Post-operative Vomiting

**Fig. 4:**
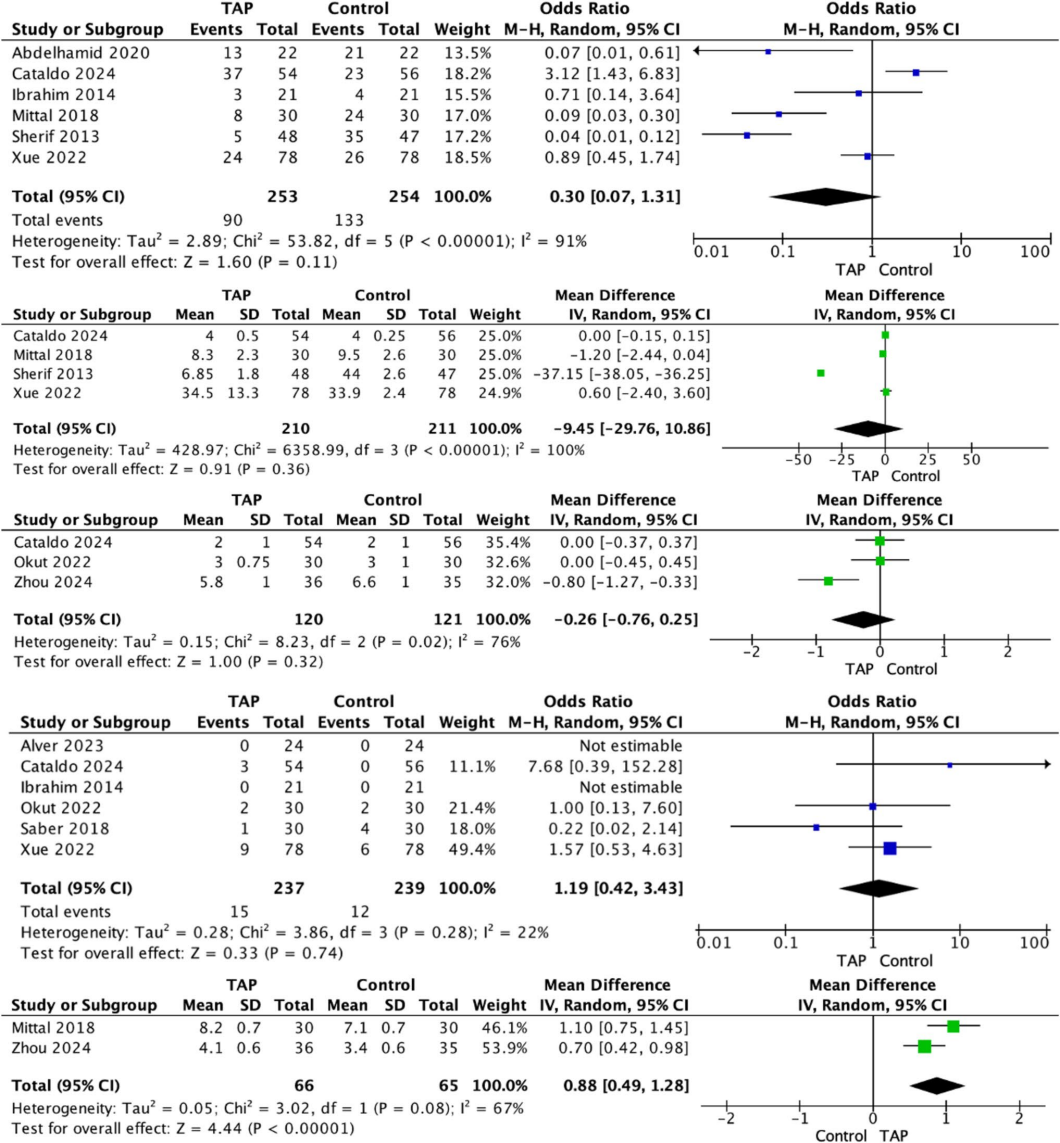
Comparison in (**A**) post-operative nausea and vomiting, **B** time to ambulation, **C** length of hospital stay, **D** complications, and (**E**) patient satisfaction scores for patients receiving transversus abdominus plane block compared to controls following laparoscopic sleeve gastrectomy

Six RCTs reported the incidence of PONV [18, 20, 22, 23, 26, 27]. Overall, 35.6% of patients receiving TAP block experienced PONV (90/253), compared to 52.4% in the control group (133/254) (*P* < 0.002, †). At meta-analysis, a non-significant difference was observed in relation to PONV for patients randomized to TAP and control respectively (OR, 0.30; 95% CI, 0.07–1.31; *P* = 0.110) (Fig. 4A).

### Time to Ambulation

Four RCTs reported the time to ambulation post-operatively [20, 23, 26, 27]. At meta-analysis, a non-significant difference was observed in relation to the time to ambulation for patients randomized to TAP and control respectively (MD, − 9.45; 95% CI, − 29.76–10.86; *P* = 0.360) (Fig. 4B).

### Length of Hospital Stay

Three RCTs reported the LOS post-operatively [20, 24, 28]. At meta-analysis, a non-significant difference was observed in relation to LOS for patients randomized to TAP and control respectively (MD, − 0.26; 95% CI, − 0.76–0.25; *P* = 0.320) (Fig. 4C).

### Complications

Six RCTs reported on post-operative complications [19, 20, 22, 24, 25, 27]. Overall, 6.3% of patients receiving TAP block developed post-operative complications (15/237), compared to 5.0% in the control group (12/239) (*P* = 0.559, †). At meta-analysis, a non-significant difference was observed in relation to post-operative complications for patients randomized to TAP and control respectively (OR, 1.19; 95% CI, 0.42–3.43; *P* = 0.740) (Fig. 4D).

### Patient Satisfaction Scores

Two RCTs reported the PSS [23, 28]. At meta-analysis, patients randomized to TAP block has higher PSS than those in the control group (MD, 0.88; 95% CI, 0.49–1.28; *P* < 0.001) (Fig. 4E).

### Risk of Bias Assessment

Risk of bias was assessed in each of the 11 prospective, randomized trials included in this review [18–28]. Five of the 11 studies were deemed to be of low and some risk of bias respectively [18–20, 22–28], while just one study was deemed to be of high risk of bias [21]. The risk of bias assessments of the included trials are outlined in detail in Supplementary Material 2.

## Discussion

This systematic review and meta-analysis of the surgical literature performed which compared the clinical efficacy of TAP block in patients living with obesity who have been indicated to undergo minimally invasive sleeve gastrectomy. This study integrated data from 776 patients who were randomized within the setting of 11 prospective, randomized trials and the results coherently demonstrate there is benefit in performing TAP blocks as an adjunct to reduce post-operative pain in the post-operative setting.

These findings suggest TAP block significantly reduces post-operative pain (as recorded using VAS at several intervals and timepoints), thus improving the subjective experience for patients undergoing sleeve gastrectomy. As one may anticipate, this study data demonstrates the most significant improvement in post-operative pain is within the first 24-h period after surgery (and TAP block administration). Pain scores became equivalent with the control group at 48 h, which may be explained by the effect of the analgesia diminishing locally, as one may expect given the half-lives of local analgesia medications rarely exceeding 24 h [29]. Importantly, this subjective reduction in post-operative pain translated directly into enhanced satisfaction rates for patients who underwent TAP block, further supporting their utility following minimally invasive sleeve gastrectomy.

Notwithstanding these important, promising findings, the results of the current study do provide some interesting and perhaps paradoxical nuances. While the use of TAP block improved subjective pain and satisfaction ratings in the post-operative setting, it failed to significantly impact the time taken to first ambulation, LOS, and post-operative complication rates following sleeve gastrectomy. The contemporary bariatric surgery paradigm has evolved such that 12-h, “same day” discharges are now anticipated and normalized for the majority of patients being managed peri-operatively in expert centers [8, 9], with ERABS protocols (including TAP block) now recommended in the second Enhanced Recovery After Surgery© Society guideline for bariatric surgery (2022), where clinically feasible [7, 30]. Given the improvements in post-operative outcomes detailed in the recent meta-analysis of prospective randomized trials [7], the administration of TAP block following sleeve gastrectomy seems a pragmatic choice when it is combined with current ERABS protocols. While reduction in subjective pain will of course remain imperative in enhancing the peri-operative journey for patients, it important to note that TAP block failed to directly improve short-term surgical outcomes in the current study. Thus, it seems that combining TAP blockade with other elements of the ERABS protocols may offer the most pragmatic means of positively influencing these outcome measures of interest.

Recent observations have highlighted the worrying trend of post-operative opioid abuse in the western world [31], with concerns that it has reached “epidemic proportions” in recent years. Accordingly, the administration of multimodal analgesic strategies, including post-operative regional anesthesia, has been promoted in contemporary practice [32], to combat the unnecessary requirement for opioid analgesia following major surgery. In the current study, TAP block administration improved VAS scores post-operatively. It failed, however, to significantly reduce breakthrough opioid analgesia consumption following surgery, while also failing to reduce the time taken for breakthrough analgesia consumption by participating patients. Therefore, while patients experienced reduced perceptions of pain, there remains a tendency to consume similar breakthrough opioid analgesia. This is concerning given that this has been noted to represent an obvious aetiology of opioid addiction within current the “opioid crisis” [33]. The authors welcome the next generation of similar RCTs to offer novel strategies to reduce the need for post-operative opioid analgesia following minimally invasive sleeve gastrectomy, in particular as the number of patients undergoing these surgeries continues to increase in the current decade [34, 35].

An important strength of this meta-analysis is that it synthesized RCT data only, which renders it to represent the zenith of scientific evidence. Importantly, the included studies are of prospective and randomized design, which means they are not subject to the inherent selection, ascertainment, and confounding biases which are apparent in studies of observational design. Despite this obvious strength, it is important to note that this study is subject to certain unavoidable limitations. Firstly, it is important to highlight there is a significant degree of heterogeneity between the RCTs included in this meta-analysis. For example, patients were recruited from six different countries from four different continents, where surgical practice and ethical obligations may vary considerably. To counteract this important issue, the authors pragmatically deployed the random effects modelling at meta-analysis to ensure such heterogeneity was accounted for within our results, irrespective of the *I*^2^ statistics outlined. Secondly, and importantly, four and two of the 11 included RCTs were from Egyptian and Turkish surgical research facilities respectively, the majority of which represent studies of considerable bias, as well as trial heterogeneity. This fails to fairly represent the application of these results to patients living with obesity across all corners of the globe, in particular, the western world. This represents an important shortcoming of this study. Thirdly, despite data being compiled from all the available RCTs in the literature specific to this topic, this meta-analysis relies on data from just 776 patients, limiting the robustness and translatability of the results yielded. Fourthly, there was an obvious disparity in induction, perioperative, and antiemetic regimens deployed in each of the studies (Supplementary Material 1). Finally, none of the included RCTs in this analysis incorporated complete (or “triple”) blinding of surgeons, patients, and statisticians into their study methodology. Accordingly, this renders this analysis subject to the reported “glass ceiling” effect evident in performing surgical RCTs, due to an apparent failure to determine the true impact of TAP block in reducing post-operative pain [36]. Such shortcomings often leads to such RCTs to be classed as “open label,” making them subject to unavoidable and unintentional biases.

In conclusion, this meta-analysis of RCTs demonstrated that the administration of TAP blocks significantly reduced post-operative pain and improved patient satisfaction following laparoscopic sleeve gastrectomy. Importantly, this reduction in pain failed to directly influence time to ambulation, LOS, and post-operative complications. Therefore, the authors do recommend that TAP should be considered in tandem with ERABS protocols in patients undergoing minimally invasive sleeve gastrectomy should expertise allow, in order to improve the patient experience in the perioperative setting.

## Supplementary Information

Below is the link to the electronic supplementary material.

Supplementary Material 1 (DOCX 21.1 KB)

Supplementary Material 2 (DOCX 16.8 KB)

## Data Availability

No datasets were generated or analysed during the current study.
