# Supplementary material for: Transversus Abdominus Plane Block for Laparoscopic Sleeve Gastrectomy—A Systematic Review and Meta-analysis of Randomized Clinical Trials
Source: Obes Surg. 2025 Sep 18;35(10):4224–33. doi: 10.1007/s11695-025-08166-z (PMC12540638; doi:10.1007/s11695-025-08166-z)
Supplement: Supplementary file 1 — Supplementary Material 1 (DOCX 21.1 KB) [file 11695_2025_8166_MOESM1_ESM.docx]

**Supplemental Material 1**. Peri-operative details from the 11 included prospective, randomised clinical trials.

| **Author** | **Year** | **General Anaesthetic Administered** | **TAP Administered** | **Surgical- or US-delivered TAP** | **Control** | **Antiemetic prescription** |
| --- | --- | --- | --- | --- | --- | --- |
| Abdelhamid | 2020 | 2 mg/kg of propofol and 0.5 mg/kg of atracurium (based on lean body weight), in addition to 100 μg of fentanyl for the induction of anesthesia | A single 30 ml shot of 0.25% bupivacaine administered as bilateral subcostal transversus abdominis plane block | US- guided (5-12 MHz) | Opioid analgesia | Patients were premedicated with 8 mg of intravenous dexamethasone and 4 mg of ondansetron. |
| Alver | 2023 | A dose of 2 mg/kg propofol, 0.5-1 μgr/kg remifentanil, and rocuronium 0.6 mg/kg IV were administered for anesthesia induction | 0.5% bupivacaine at a dose of 1.5 mg/kg for block procedure. A diluted 0.5% bupivacaine at lower concentrations with normal saline was used. A mixture of bupivacaine and saline was used. After confirmation, 40 ml of local anesthetic solution was given bilaterally to the interfascial plane between transversus abdominis and internal oblique muscles. | US-guided (8-12 MHz) | Local anaesthetic into port sites | N/R |
| Cataldo | 2024 | GA was induced through the administration of propofol 2.5–3 mg/Kg for adjusted body weight (ABW) to obtain a bispectral index (BIS) between 40 and 60, fentanyl 200 mcg, and rocuronium 1 mg/ABW to obtain a deep neuro- muscular block | LG-TAP block with LA combined with LA-PSI (LG-TAP group) - Infiltration of the laparoscopic access using a total of 10 ml ropivacaine 0.5% | Laparoscopic- guided at the beginning of procedure | 0.9% saline solution combined with port-site infiltration | Antiemetic prophylaxis with dexamethasone 8 mg, and granisetron 3 mg, |
| Hussein | 2023 | IV fentanyl (1–2 g/kg) and propofol (2 mg/kg) to produce general anaesthesia | 0.2 mL/kg of 0.25% bupivacaine | US-guided | Quadratus lumborum block | IV granisetron (1 mg) as antiemetic |
| Ibrahim | 2014 | Propofol (2.5 mg/kg), fentanyl (2 mcg/kg), and cisatracurium (0.15 mg/kg), with intraoperative non-opioid analgesia of paracetamol (15–20 mg/kg) Lornoxicam (zefoR, NYCOMED Austria) 8 mg slowly IV. | Bilateral TAP block using a standardized dose of 30ml of bupivacaine hydrochloride 0.25% using (Marcain, Astra Zeneca, UK) in each side plus port site infiltration of 20 ml sterile normal saline. | US-guided (7-13 MHz) | Placebo group received TAP block and port site infiltration by same volumes of sterile normal saline | Dexamethasone 8 mg IV, and ondansetron 4 mg IV for antiemetic prophylaxis. |
| Mittal | 2018 | Standard general anesthesia technique with endotracheal intubation and muscle paralysis. | US-guided injection of 40 ml of 0.375% ropivacaine was injected in the fascial plane and was observed to spread between the two layers on either side for a bilateral TAP block. | US-guided (7-12 MHz) | Only systemic analgesia | N/R |
| Okut | 2022 | N/R | 20 ml of 5% bupivacaine, diluted with 30 ml of saline, was injected bilaterally in equal amounts into the plane between the internal oblique and transverse abdominis muscles | Laparoscopic- guided | None | N/R |
| Saber | 2018 | Propofol, midazolam, and fentanyl were given in a weight based dosing. | TAP block with 0.25% bupivacaine (40mL total) | US-guided | Placebo | Intravenous metoclopramide 10mg and dexamethasone 8mg, with Ondansetron 4mg given prior to extubation |
| Sherif | 2013 | Fentanyl (1–2 μg/kg), propofol (2–3 mg/kg), and rocuronium (0.6 mg/kg) | 20 ml of 0.5% bupivacaine was injected into the patients of the TAP block group | US-guided (5-12 MHz) | None | Ondansetron 4 mg was prescribed for nausea and vomiting prophylaxis. |
| Xue | 2022 | General anesthesia was induced with midazo- lam 0.025 mg kg-1, propofol 1.5 mg kg-1, cisa- tracurium 0.2 mg kg-1, and remifentanil 2 lg kg-1. | 30 ml of 0.33% ropivacaine (including dexmedetomidine 1 lg kg-1) | US-guided (2-5 MHz) | GA alone | 3 mg granisetron, as prophylaxis against PONV was administered. |
| Zhou | 2024 | Midazolam (0.05mg/kg), propofol (1–2 mg/kg, total body weight), and rocuronium (0.6 mg/kg) | Bilateral TAP block procedures (ropivacaine 0.3%, 20mL) guided by ultrasound | US-guided | opioid-based anesthesia group | Ondansetron (8mg i.v) and droperidol (10~15μg/kg i.v) were administered |

TAP; Transversus Abdominus plane block, US; ultrasound, N/R; not reported, GA; general anesthetic, IV; intravenous, PONV; postoperative nausea and vomiting
