# Supplementary material for: Transversus Abdominus Plane Block for Laparoscopic Sleeve Gastrectomy—A Systematic Review and Meta-analysis of Randomized Clinical Trials
Source: Obes Surg. 2025 Sep 18;35(10):4224–33. doi: 10.1007/s11695-025-08166-z (PMC12540638; doi:10.1007/s11695-025-08166-z)
Supplement: Supplementary file 2 — Supplementary Material 2 (DOCX 16.8 KB) [file 11695_2025_8166_MOESM2_ESM.docx]

**Supplemental Material 2**. Detailed risk of bias assessment breakdown from the 11 included prospective, randomised clinical trials.

| **Author** | **Year** | **RSG** | **AC** | **BOP** | **BOOA** | **IOD** | **SR** | **OB** | **Overall** |
| --- | --- | --- | --- | --- | --- | --- | --- | --- | --- |
| Abdelhamid | 2020 | Low | Some | Low | Some | Low | Low | Some | Some |
| Alver | 2023 | Some | Low | High | Some | Low | Low | Low | Some |
| Cataldo | 2024 | Low | Low | Low | Low | Some | Low | Low | Low |
| Hussein | 2023 | Some | Some | High | High | Low | Some | Some | High |
| Ibrahim | 2014 | Low | Some | Low | Low | Some | Some | Low | Some |
| Mittal | 2018 | Low | Some | Low | Some | Some | Low | Some | Some |
| Okut | 2022 | Some | Some | Low | Low | Some | Some | Low | Some |
| Saber | 2018 | Some | Low | Low | Low | Low | Low | Low | Low |
| Sherif | 2013 | Some | Low | Low | Low | Low | Low | Low | Low |
| Xue | 2022 | Some | Some | Low | Low | Low | Low | Low | Low |
| Zhou | 2024 | Low | Low | Low | Low | Low | Low | Some | Low |

RSG; random sequence generation, AC; allocation concealment,
BOP; blinding of participants, BOOA; blinding of outcome assessment,
IOD; incomplete outcome data, SR; selective reporting, OB; other bias
